# Familiarity mediates apes' attentional biases toward human faces

**DOI:** 10.1098/rspb.2021.2599

**Published:** 2022-04-27

**Authors:** Jesse G. Leinwand, Mason Fidino, Stephen R. Ross, Lydia M. Hopper

**Affiliations:** ^1^ Lester E. Fisher Center for the Study and Conservation of Apes, Lincoln Park Zoo, Chicago, IL, USA; ^2^ Urban Wildlife Institute, Lincoln Park Zoo, Chicago, IL, USA; ^3^ Department of Molecular and Comparative Pathobiology, Johns Hopkins University School of Medicine, Baltimore, MD 21205, USA

**Keywords:** cognitive bias, dot-probe, emotion, familiarity, novelty, recognition

## Abstract

In zoos, primates experience markedly different interactions with familiar humans, such as the zookeepers who care for them, compared with those with unfamiliar humans, such as the large volume of zoo visitors to whom they are regularly exposed. While the behaviour of zoo-housed primates in the presence of unfamiliar, and to a lesser extent familiar, humans has received considerable attention, if and how they spontaneously distinguish familiar from unfamiliar people, and the cognitive mechanisms underlying the relationships they form with familiar and unfamiliar humans, remain poorly understood. Using a dot-probe paradigm, we assessed whether primates (chimpanzees and gorillas) show an attentional bias toward the faces of familiar humans, with whom the apes presumably had a positive relationship. Contrary to our predictions, all subjects showed a significant attentional bias toward unfamiliar people's faces compared with familiar people's faces when the faces showed a neutral expression, both with and without a surgical face mask on, but no significant attentional bias when the faces showed a surprised expression. These results demonstrate that apes can spontaneously categorize humans based on familiarity and we argue that the attentional biases the apes showed for unfamiliar human faces reflect a novelty effect.

## 1. Introduction

Humans have a longstanding interest in wild and exotic animals [1], as evidenced by the 700 million people who visit zoos around the world every year [2]. Given this, and in contrast to most animals housed in other captive settings, the lives of zoo animals are defined by regular exposure to large numbers of unfamiliar humans (i.e. zoo visitors) in addition to familiar humans (i.e. care staff) [3]. Although zoo-housed animals see, and potentially interact with, a variety of people daily, there is likely to be substantial variation in the quality of human–animal relationships (HARs) that animals form with different categories of people (e.g. caretakers, veterinarians, visitors) as well as with different individuals within those categories [4]. Hosey [5] theorized that the repeated positive interactions animals have with familiar people result in positive HARs, as compared with unfamiliar people, with whom animals likely form generalized neutral or negative HARs. Supporting this, nonhuman primates show contrasting responses to different people based on how those people behave and interact with them (e.g. [6–9]), but what is less well understood is whether primates differentiate *categories* of people, specifically familiar from unfamiliar people.

A number of studies have evaluated the effects of visitors on the behaviour of zoo-housed nonhuman primates (primates hereafter) (e.g. [10–16]) and the behaviour of zoo-housed primates in the presence of familiar humans (e.g. [10,11,17–20]). Moreover, recent research has demonstrated that several primate species show differential behavioural responses to familiar versus unfamiliar people [10,11,21]. Sanctuary-housed bonobos (*Pan paniscus*), for instance, not only appear to take humans' attentional states into account but modify their communication signals toward humans based on their familiarity with that person, repeating previously successful signals more often with familiar than unfamiliar people [22]. Similarly, Smith [11] reported that gorillas (*Gorilla gorilla gorilla*) and orangutans (*Pongo abelii*) begged from familiar zoo staff far more often than from unfamiliar zoo visitors, and the apes engaged in more visual monitoring of familiar than unfamiliar people. This suggests that primates differentiate people based on familiarity, although the context and people's behaviour may cue the primates' responses in such cases. Furthermore, relying on behavioural observations as a measure of attention may be unreliable owing to the brief and subtle nature of such responses [23]. Thus, more precise measures of primates’ attention are required to assess their ability to discriminate familiar from unfamiliar people, and what identity information they use to do so.

As humans can discriminate familiar from unfamiliar (or novel) faces [24–27], primates may also be differentially sensitive to the faces of familiar and unfamiliar people. A number of studies have examined primates' ability to differentiate familiar and unfamiliar conspecifics [28–33], and their ability *to learn to* discriminate between different images of human faces [34–36]. However, there is minimal research regarding whether primates *spontaneously* differentiate heterospecifics (especially humans) based solely on familiarity [37]. Using an emotional Stroop task, Allritz and colleagues [38] reported that zoo-housed chimpanzees (*Pan troglodytes*) were slower to touch photographs of the zoo veterinarian, as compared with images of a familiar caretaker or an unfamiliar person, suggesting an influence of familiarity and valence on the apes’ responses. In a separate study examining the interplay between familiarity and emotional valence, van Berlo *et al*. [39] found that bonobos did not show a significant attentional bias toward familiar compared with unfamiliar human faces, regardless of whether the faces were neutral or expressed emotion. These bonobos did, however, show a significant attentional bias toward emotional scenes of unfamiliar, but not familiar, conspecifics.

Building on the study of van Berlo *et al*. [39], we sought to assess whether chimpanzees and gorillas would spontaneously recognize photographs of multiple familiar people and differentiate them from photographs of multiple unfamiliar people. To do so, we used a dot-probe task [40,41] to explore zoo-housed apes' potential attentional biases toward different people based on familiarity (see the electronic supplementary material for additional information on the dot-probe paradigm). After validating the paradigm by presenting a photograph of a human's face alongside a pixelated version of the same photograph to test the apes' differential attention to unaltered and pixelated faces, we ran a series of three experiments to examine zoo-housed apes’ spontaneous responses to familiar and unfamiliar people (see the electronic supplementary material for additional information on the pretest method validation and results).

In our first experiment, (1) we paired photographs of familiar and unfamiliar humans' faces with neutral expressions to test the apes’ spontaneous ability to discriminate human faces based on familiarity. While this has not explicitly been tested before in chimpanzees or gorillas, we predicted that the apes would show a differential response to familiar as compared with unfamiliar faces, given that other species (e.g. dogs (*Canis familiaris*) [42], sheep (*Ovis aries*) [43] and horses (*Equus caballus*) [44]) have been shown to discriminate between familiar and unfamiliar humans, although often only after training. Moreover, following Hosey [5], and in line with research showing that humans demonstrate a familiarity preference for faces [45,46], we predicted that the apes would show an attentional bias toward familiar people's faces. As previous research with bonobos has found that attention to emotional conspecific faces, but not necessarily emotional human faces, is influenced by familiarity [39], in our second experiment, (2) we paired familiar and unfamiliar human faces that showed a surprised expression. We chose surprise because it is a visibly salient emotion that the apes likely observe less frequently, but that nonetheless clearly alters the appearance of multiple facial features and is one that, in humans at least, is typically correctly recognized [47]. Given the limited and mixed data for *Pan* [39,41,48,49], we did not have a directional prediction for how the apes would respond to these images. Lastly, (3) we paired photographs of familiar and unfamiliar individuals wearing a blue surgical face mask (with a neutral expression). We selected a face mask as a way of creating ‘real world’ images that limited the amount of identifying information provided to the apes, without relying on image manipulation, to increase stimuli validity. This was done as these apes have had extended and regular interactions with familiar people (their care staff) wearing surgical face masks, but not unfamiliar people; although the apes had limited experience viewing unfamiliar people (e.g. zoo visitors) wearing masks due to the COVID-19 pandemic (see the electronic supplementary material for additional details about the apes' relative exposure to people wearing masks, including during the COVID-19 pandemic). As with our first experiment, we predicted that the apes would show an attentional bias toward familiar faces, even with reduced identifiable information available to them.

## 2. Methods

### (a) Subjects and housing

We tested 12 zoo-housed great apes: seven chimpanzees (4 females, 3 males, average age: 25.47 years, s.d. = 9.08) and five male gorillas (average age: 19.13, years, s.d. = 7.44) (electronic supplementary material, table S1). All 12 apes lived in social groups at Lincoln Park Zoo (Chicago, USA) and had experience using touchscreens prior to the start of this study. For more information about the subjects and housing, see the electronic supplementary material.

### (b) General testing protocol

**Figure 1.**
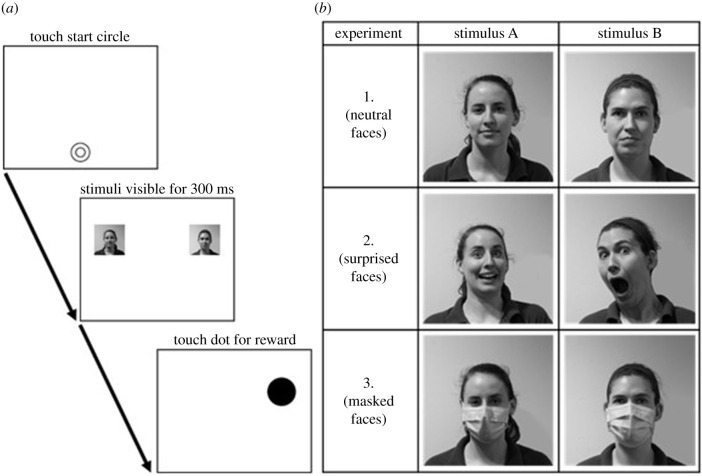
(*a*) Example trial design from experiment 1 showing a familiar face and an unfamiliar face, both in greyscale with a neutral expression. Across trials, the locations of the familiar and unfamiliar faces were counterbalanced, as was the location of the dot that replaced the photographs. (*b*) Examples of the pairs of stimuli for the three experiments. In experiment 1 a photograph of a familiar person's face was paired with a photograph of an unfamiliar person's face, both with neutral expressions; in experiment 2 a photograph of a familiar person's face was paired with a photograph of an unfamiliar person's face, both with surprised expressions; and in experiment 3 a photograph of a familiar person's face was paired with a photograph of an unfamiliar person's face, both with neutral expressions while wearing a surgical face mask.

Across all three experiments, described in detail below and in the electronic supplementary material, our general approach to testing the subjects remained consistent. We tested all subjects using 10-point 55 cm capacitive ViewSonic LCD touchscreen monitors (1920 × 1080 resolution) using Zenrichment ApeTouch software v. 14.4 [50]. We used the dot-probe attentional bias paradigm [40] for all three experiments in this study. Each trial began with a black start circle (210 pixel diameter) centrally located at the bottom of a white screen. This was done to both focus the subject's attention to the screen and centre their hand prior to each trial. Upon touching the start dot, it disappeared and two different lateralized stimuli (each 400 × 400 pixels) appeared on the screen. Following Kret *et al*. [41], these two stimuli were visible for 300 ms—long enough that the subjects could consciously process them [51], but not long enough for them to alternate their gaze back and forth between the two stimuli. After 300 ms, both stimuli disappeared and a black dot (300 pixel diameter) replaced one of them (figure 1*a*). The subjects could then touch this dot, which would result in a ‘chime’ sound they were familiar with from previous tasks as signifying a correct response. Once they touched the black dot, we gave them a food reward (one blueberry). There was no time limit for subjects to touch the dot. We employed a 4 s intertrial interval (ITI) to allow subjects time to collect and eat their reward between trials, at which point the start dot would reappear and the subject could initiate the next trial. Thus, subjects were rewarded for every trial, and response latency was our dependent variable. Data for all three experiments were collected from May 2021 through August 2021.

### (c) Stimuli

#### (i) Experiment 1: neutral familiar versus neutral unfamiliar faces

We used photographs of nine familiar and nine unfamiliar human faces as stimuli. Familiar individuals were members of the apes' care staff with a minimum of six months of experience working directly with the apes. Additionally, all familiar individuals routinely interacted with all the subjects via protected contact (e.g. safely separated from the apes by a physical barrier; steel mesh or glass viewing windows). Unfamiliar individuals were zoo employees from other departments who never interacted with the apes (e.g. members of the zoo's horticulture team). All individuals, both familiar and unfamiliar, wore identical Lincoln Park Zoo green polo shirts for the pictures for consistency, although only the shirt collar and tops of shoulders were visible in the pictures. We photographed all the people via identical methods (described in the electronic supplementary material) and we converted all images to greyscale for presentation to the apes (figure 1*b*).

#### (ii) Experiment 2: surprised familiar versus surprised unfamiliar faces

We used photographs of the same nine familiar humans and the same nine unfamiliar humans for experiment 2 as were photographed for experiment 1, with one photograph per person. However, for experiment 2, we asked the people to look ‘surprised’ for these photographs (figure 1*b*). As with the previous experiment, all photographs were presented in greyscale.

#### (iii) Experiment 3: masked familiar versus masked unfamiliar faces

We again used photographs of the same nine familiar humans and nine unfamiliar humans for experiment 3 as were used in experiments 1 and 2. However, here we photographed the familiar and unfamiliar humans with neutral expressions while they were wearing a single blue surgical face mask (figure 1*b*). As with the previous experiments, all photographs were presented in greyscale.

### (d) Procedure

Across all three experiments, our procedures remained the same, with the different stimuli being the only change. For each trial, subjects were presented with two photographs, one of a familiar face and one of an unfamiliar face. To control for variation among individual faces and expressions, trials were fully counterbalanced and pseudo-randomized such that subjects experienced one trial of each possible configuration of the task. That is, each of the nine familiar faces was paired with each of the nine unfamiliar faces four times such that the familiar face could be on the right or left side of the screen and the dot could replace the familiar or unfamiliar face. This resulted in 324 different configurations (i.e. trials), which we pseudo-randomly divided across nine 36-trial sessions per subject. We also ensured: that the same type of stimulus (familiar or unfamiliar) never appeared more than three consecutive times on the same side of the screen; that the dot never appeared more than three consecutive times on the same side of the screen; and that the dot never replaced the same type of stimulus (familiar or unfamiliar) on more than three consecutive trials.

### (e) Data analysis

Following Lacreuse *et al*. [52], we prepared our data for analysis by first removing all trials in which the subject's response latency was greater than 1000 ms, for which it might be presumed that the subject was distracted or some other experimental error arose. Doing so resulted in the removal of 461 of 3888 trials (11.86%) for experiment 1, 384 of 3888 trials (9.88%) for experiment 2, and 487 of 3888 trials (12.53%) for experiment 3 (see electronic supplementary material, table S1 for the number of trials analysed per subject). We note that Lacreuse *et al*. [52] also trimmed their data to remove all trials with very short response latencies (i.e. less than 100 ms), but we did not find any trials in our dataset for which this was the case. All raw data and R scripts are provided in the electronic supplementary material.

As our data were strictly positive (latencies can only be greater than 0 ms) and right-censored (i.e. we excluded all responses with a latency greater than 1000 ms), we ran a survival analysis for the response variable *latency* (ms) *to touch* as our measure of attentional bias (*sensu* [53], see also [54,55]). For all experiments, we fitted Cox proportional hazards regression mixed-effects models using the *coxph* function of the *survival* library [56] in R v. 4.1.0 [57] to explore the relative importance of Species and Stimuli Type on the subjects' response latencies (*sensu* [53]). This class of model is a form of survival analysis [58] that allows the incorporation of categorical variables under a regression modelling framework, making it an ideal choice for our data and research questions. We then examined Familiarity (familiar face, unfamiliar face), Species (chimpanzee, gorilla), and potential interaction effects between them for each experiment. This resulted in five models (four planned contrasts plus a null model) for each experiment. We included individual subject IDs as a random effect in each model to control for differences in response latency among individuals.

For each experiment, we used Akaike's information criterion (AIC) values for model selection and considered all models within 2ΔAIC of the best-fit model as competitive [59]. To evaluate evidence of an effect from the best-fit models of each experiment, we used a bootstrap resampling procedure of the data 1000 times. For each bootstrap and experiment, we split the data for each individual into individual datasets, randomly resampled each individual's respective data with replacement, and then combined all of the resampled individual data to generate a dataset with the same number of samples from each individual. This resampling procedure was done to ensure that each individual was present in the bootstrapped dataset, which was necessary to fit the random effect term in the model. Following this, we refitted the best-fit model to the resampled data and collected the parameter estimates from each bootstrap. Doing so generated a distribution of parameter estimates from the best-fit model, which we used to construct 95% confidence intervals for each parameter. We determined evidence of an effect if the 95% confidence intervals of an associated parameter did not overlap zero.

## 3. Results

### (a) Experiment 1: neutral familiar versus neutral unfamiliar faces

**Figure 2.**
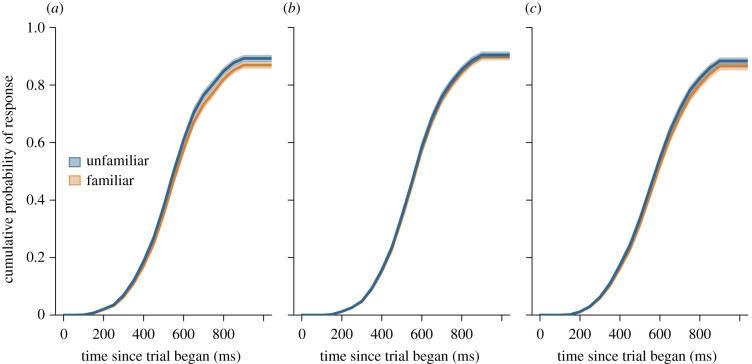
The cumulative probability apes select either familiar or unfamiliar faces during each experimental trial, on average. Here, ‘time since trial began’ represents the time taken for the apes to touch the black dot on the screen after it appeared, replacing one of the two photographs. (*a*) In experiment 1, the apes were significantly faster to touch the dot when it replaced an unfamiliar face showing a neutral expression as compared with when it replaced a familiar face also showing a neutral expression. (*b*) In experiment 2, the apes did not show a significant difference in response latency when the dot replaced an unfamiliar face showing a surprised expression as compared with when it replaced a familiar face also showing a surprised expression. (*c*) In experiment 3, the apes were significantly faster to touch the dot when it replaced an unfamiliar face wearing a surgical face mask as compared with when it replaced a familiar face also wearing a surgical face mask. Thick lines represent the mean response latency among apes, while the shaded areas show 95% confidence intervals.

**Figure 3.**
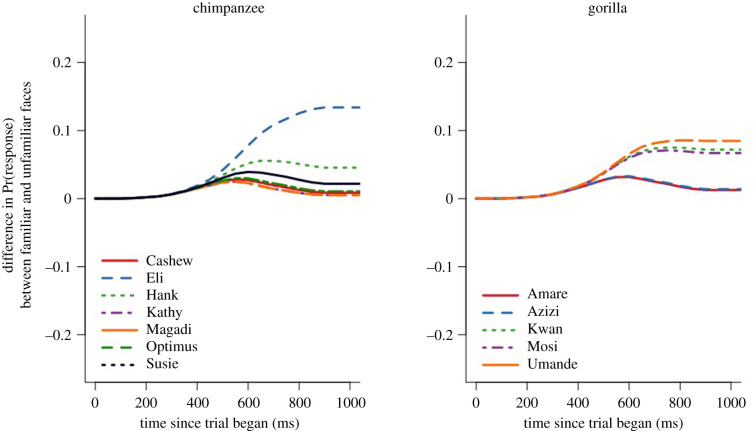
Individual subject results for experiment 1. Lines above 0 indicate that subjects responded faster to unfamiliar neutral faces than familiar neutral faces—a pattern that all apes showed. Here, ‘time since trial began’ represents the time taken for the apes to touch the black dot on the screen after it appeared, replacing one of the two photographs. 95% CIs are excluded for clarity.

Of the five models fitted to the data for this experiment, three models were considered competitive (electronic supplementary material, table S4). All three models included the factor of Familiarity. Although the first two candidate models also included Species, given the results our pretest (i.e. that there was an overall species difference in the response speeds of chimpanzees and gorillas, such that gorillas responded more slowly than chimpanzees, but no species difference as to *how* they responded to different categories of stimuli; electronic supplementary material, table S2 and figure S2), we selected the model that just included Familiarity as the best candidate (*sensu* [60]). This model revealed that there was a significant effect of Familiarity, such that the apes were significantly quicker to respond in trials in which the dot replaced unfamiliar as opposed to trials in which it replaced familiar human faces (*χ*^2^ = 9.93, s.e.^2^ 0.03, d.f. = 1.00, *p* < 0.001) (i.e. the apes showed an attentional bias toward unfamiliar people's faces) (figure 2). We found that, on average, subjects were approximately 11% (95% CI = 3%, 20%) slower to touch the dot when it replaced a familiar face compared with an unfamiliar face. While all subjects showed the same pattern of responses (figure 3), chimpanzee Eli showed the most pronounced pattern in his responses, responding even more quickly to unfamiliar faces relative to familiar faces as compared with the other apes.

### (b) Experiment 2: surprised familiar versus surprised unfamiliar faces

For experiment 2, we found that the null hypothesis was competitive, indicating that there were no significant main effects or interaction effects for Species or Familiarity (electronic supplementary material, table S5). Although not a significant effect, we found that the apes were approximately 4% (95% CI = −3%, 11%) slower to touch the dot when it replaced a familiar face compared with an unfamiliar face (figure 2), following the apes' pattern of responses in experiment 1. We saw little individual variation in this experiment, with all subjects showing similar patterns in their responses (electronic supplementary material, figure S4).

### (c) Experiment 3: masked familiar versus masked unfamiliar faces

Of the five models fitted to the data for experiment 3, two models were considered competitive (electronic supplementary material, table S6). Both candidate models included the factors of Familiarity and Species, although the best-fit model did not include an interaction between these factors. The best-fit model revealed that there was a significant main effect for Familiarity, such that the apes were significantly quicker to respond when the dot replaced unfamiliar as opposed to familiar faces (*χ*^2^ = 6.60, s.e.^2^ 0.03, d.f. = 1.00, *p* = 0.01) (figure 2). We found that subjects were, on average, 9% (95% CI = 2%, 17%) slower to touch the dot when it replaced a familiar face compared with an unfamiliar face. We also found a significant main effect for Species, such that the chimpanzees were, on average, 44% (95% CI = 40%, 48%) quicker than the gorillas to respond in trials (*χ*^2^ = 11.12, s.e.^2^ 0.04, d.f. = 1.00, *p* < 0.001). This reflects the species difference in overall response latencies we saw in the pretest (electronic supplementary material, figure S2), a pattern we find across all touchscreen tasks these apes have completed, in which the larger-bodied gorillas are slower to touch the screen than the smaller-bodied chimpanzees. We saw little individual variation in this experiment, with all subjects showing similar patterns in their responses (electronic supplementary material, figure S5).

## 4. Discussion

We sought to assess whether chimpanzees and gorillas would show differential attentional biases to the faces of familiar versus unfamiliar people and whether their responses were mediated by image valence (neutral or surprised expressions), or the amount of identity information provided (unmasked or masked faces). The apes exhibited significant attentional biases toward the emotionally neutral faces of unfamiliar humans compared with those of familiar humans—even when the faces were partially occluded by a surgical face mask. This pattern was consistent for all subjects that we tested. When the apes were shown faces with a surprised expression, they showed a more muted response: while they showed a pattern of responding faster to unfamiliar than familiar faces, this difference was not statistically significant. The apes’ responses suggest that they can classify people based on familiarity, from their faces alone. Importantly, the condition of familiarity was not generated within the testing protocol via habituation effects, but rather we used ‘real world’ stimuli from the apes' environment (*sensu* [30,37,39]). Specifically, the familiar faces were those of the apes’ care staff, each of whom had a minimum of six months' experience routinely interacting with the apes and, likely, with whom the apes had formed a relationship. However, and contrary to Hosey's HAR hypothesis [5], the apes directed their attention toward unfamiliar people, not their familiar care staff. These results show not only that the apes spontaneously differentiated people with whom they had a relationship over people they did not, but that they made these distinctions rapidly (the stimuli were only presented for 300 ms), from two-dimensional greyscale photographs, and from only viewing the person's face, without behavioural or other context cues, or any training. Why, though, did the apes show an attentional bias toward unfamiliar, rather than familiar, people as we had predicted?

We suggest that the apes' responses reflect a novelty effect [61,62], whereby individual's attention is captured by novel stimuli, owing to either curiosity or threat detection [63]. Recent behavioural research with zoo-housed apes suggests that captive apes are curious, rather than cautious, about unfamiliar people [10–12]. We believe that curiosity is more likely driving this effect than threat detection, although it is important to note that measuring solely gaze or attention does not allow us to differentiate between caution and interest [11]. Nonetheless, it remains plausible that unfamiliar heterospecifics could constitute a potential threat or risk. Indeed, when shown *conspecific* faces, apes show an attentional bias for emotionally valent unfamiliar individuals [39] and dominant familiar individuals [30], which may reflect a drive to attend to socially important information. Similarly, chimpanzees have been shown to look longer at and scan novel conspecific faces more extensively than familiar faces [33]. However, given the evidence that apes also preferentially attend to images of familiar over unfamiliar conspecifics in emotionally neutral contexts [30], more research is needed to disentangle the relative influence of familiarity, emotional valence and species on apes' attentional biases and what that can tell us about how apes value and categorize different social information. This need for additional research is apparent from our finding that the apes showed an attentional bias toward the faces of unfamiliar heterospecifics, yet this effect was not significant when the people had emotionally valent (e.g. surprised) expressions. We do not have a clear explanation as to why familiarity did not mediate the apes’ response to surprised faces in experiment 2; however, given that the apes again showed a significant attentional bias toward unfamiliar compared with familiar people's faces wearing surgical masks in experiment 3, we do not believe that our results in experiment 2 reflect a habituation effect to the task. Rather, the lack of differential response may reflect an interaction effect between familiarity, emotional valence, and species [39]. Alternatively, it is possible that the novelty of seeing a surprised expression on familiar faces made them appear unfamiliar, or the surprised expression itself may have distorted the features of even familiar faces sufficiently to render them less recognizable.

Our study provides more detailed evidence that apes spontaneously discriminate and categorize human faces based on familiarity. Cognitive and neurological research has demonstrated that a novelty preference appears hardwired in human infants for objects [64], and that infant attention is also biased toward novel human faces [65]. However, research with adult humans has found that context often determines whether familiarity or novelty drive visual attentional biases [66,67] and preferences [45,46]. For example, adults show a familiarity preference for human faces, but a novelty preference for natural scenes [45,46]. Intriguingly, for macaques, it appears that novelty and value coding are linked, with both ‘novel’ and ‘good’ objects activating some of the same neural networks [68]. Moreover, neuroimaging studies suggest that not only are different brain regions responsible for the perception of certain classes of stimuli, such as faces and natural scenes [69], but different neural pathways are responsible for how humans [24–27] and primates [70,71] process familiar and unfamiliar faces. Accordingly, while certain innate attentional biases may be present in both humans and primates, rearing history, learning and other social and environmental factors invariably influence attention as well [72]. Having all been reared in captive settings, the apes we tested were exposed to large numbers of (unfamiliar) humans throughout their lives without negative consequences, and thus likely did not view unfamiliar humans as threats. Therefore, we argue that stimulus novelty likely mediated their responses.

Due to the potential implications of understanding captive primates' (and other species’) attentional biases to further enhance their welfare, researchers are increasingly using cognitive and attentional bias paradigms with captive animals [73,74]. Given the unique aspects of zoos (i.e. the presence of many unfamiliar people), our study helps shed new light on zoo-housed primates' experiences and adds to a growing effort to better understand animal wellbeing using methods beyond standard behavioural measures [73–75]. Attentional bias research thus has the potential to assess both HARs and welfare (e.g. [75,76]) by focusing on what have been termed affect-driven attentional biases [73], which refer to the stimuli animals attend to as modulated by their affective states. As attentional biases can reflect the affective state of an animal, measuring these biases provides valuable welfare indicators [73]. Moreover, primates (and other species) in other captive settings, such as sanctuaries and research facilities, also encounter both familiar and unfamiliar people (e.g. [77]), among other stimuli, so the value of attentional bias research paradigms as a tool to assess and monitor the affective states, and therefore welfare, of animals extends beyond zoo settings.

## 5. Conclusion

Contrary to our hypothesis based on Hosey's HAR model [5], the chimpanzees and gorillas showed attentional biases toward the faces of unfamiliar people as compared to the faces of familiar humans. Nonetheless, the homogeneity of the apes' responses demonstrates that all subjects differentiated their familiar care staff from unfamiliar people and further highlights the validity of these results and the viability of the dot-probe paradigm for assessing primate attentional biases and welfare [73]. While we predicted that the apes’ attention would be biased toward the faces of their care staff with whom they had already formed relationships, we instead found evidence suggestive of a novelty effect. However, our results do not negate Hosey's model [5] because our dot-probe paradigm assessed what initially captured the apes' attention rather than what the apes preferred or fixated on, which may better reflect preferences (and HARs). Future research may seek to further explore what mediates primates’ responses to familiar people, such as the length, strength and valence of their relationship with them [38] to better understand what attentional bias studies can tell us about HARs. Varying the presentation length of the stimuli and using an eye-tracker may bolster such research by allowing for a more nuanced analysis of attentional allocation to the specific features of these stimuli [23].

## Data Availability

Code and data are provided as electronic supplementary material [78].
